# Assessment of two malaria rapid diagnostic tests in children under five years of age, with follow-up of false-positive pLDH test results, in a hyperendemic falciparum malaria area, Sierra Leone

**DOI:** 10.1186/1475-2875-9-28

**Published:** 2010-01-21

**Authors:** Sibylle Gerstl, Sophie Dunkley, Ahmed Mukhtar, Martin De Smet, Samuel Baker, Jacob Maikere

**Affiliations:** 1 Médecins Sans Frontières (MSF UK), 67-74 Saffron Hill, London EC 1N 8QX, UK; 2 Médecins Sans Frontières (MSF OCB), Bo-Kenema Highway, Bo, Sierra Leone; 3 Médecins Sans Frontières, 94, rue Dupré, 1090 Brussels, Belgium; 4 Ministry of Health and Sanitation, Freetown, Sierra Leone

## Abstract

**Background:**

Most malaria rapid diagnostic tests (RDTs) use HRP2 detection, including Paracheck-Pf^®^, but their utility is limited by persistent false positivity after treatment. PLDH-based tests become negative more quickly, but sensitivity has been reported below the recommended standard of 90%. A new pLDH test, CareStart™ three-line P.f/PAN-pLDH, claims better sensitivity with continued rapid conversion to negative. The study aims were to 1) compare sensitivity and specificity of CareStart™ to Paracheck-Pf^® ^to diagnose falciparum malaria in children under five years of age, 2) assess how quickly false-positive CareStart™ tests become negative and 3) evaluate ease of use and inter-reader agreement of both tests.

**Methods:**

Participants were included if they were aged between two and 59 months, presenting to a Médecins Sans Frontières community health centre in eastern Sierra Leone with suspected malaria defined as fever (axillary temperature > 37.5°C) and/or history of fever in the previous 72 hours and no signs of severe disease. The same capillary blood was used for the RDTs and the blood slide, the latter used as the gold standard reference. All positive participants were treated with supervised artesunate and amodiaquine treatment for three days. Participants with a persistent false-positive CareStart™, but a negative blood slide on Day 2, were followed with repeated CareStart™ and blood slide tests every seven days until CareStart™ became negative or a maximum of 28 days.

**Results:**

Sensitivity of CareStart™ was 99.4% (CI 96.8-100.0, 168/169) and of Paracheck-Pf^®^, 98.8% (95% CI 95.8-99.8, 167/169). Specificity of CareStart™ was 96.0% (CI 91.9-98.4, 167/174) and of Paracheck-Pf^®^, 74.7% (CI 67.6-81.0, 130/174) (p < 0.001). Neither test showed any change in sensitivity with decreasing parasitaemia. Of the 155 eligible follow-up CareStart™ participants, 63.9% (99/155) had a false-positive test on day 2, 21.3% (33/155) on day 7, 5.8% (9/155) on day 14, 1.9% (3/155) on day 21 and 0.6% (1/155) on day 28. The median time for test negativity was seven days. CareStart™ was as easy to use and interpret as Paracheck-Pf^® ^with excellent inter-reader agreement.

**Conclusions:**

Both RDTs were highly sensitive, met WHO standards for the detection of falciparum malaria monoinfections where parasitaemia was >100 parasites/μl and were easy to use. CareStart™ persistent false positivity decreased quickly after successful anti-malarial treatment, making it a good choice for a RDT for a hyperendemic falciparum malaria area.

## Background

Prompt and accurate diagnosis followed by effective treatment is currently the main malaria control strategy besides preventive measures. Widespread documented resistance to the older common anti-malarial mono-therapies, notably chloroquine, has led to an increased use of the highly effective artemisinin-based combination therapy (ACT) as the first-line treatment for uncomplicated *Plasmodium falciparum *malaria [1,2].

Accurate confirmation of malaria diagnosis can reduce the overuse of ACT treatment and, therefore, delay the development of resistance. Moreover, it may both reduce the risk of adverse drug reactions due to unnecessary treatment and increase correct treatment for pathologies other than malaria. This, in turn, increases patient and parents/caretakers confidence in health services by providing accurate information for a proper diagnosis [3,4]. Currently, the most commonly accepted gold standard diagnostic method is microscopic reading of a stained blood film. This requires laboratory technicians with training and technical expertise, good quality reagents, a well-maintained microscope and is time consuming. From experience it is challenging to have microscopic results in less than two hours, as recommended by WHO, even with well-trained staff. In many populations where malaria is prevalent, no laboratory facilities and/or trained staff are present, making access to microscopically confirmed diagnosis impossible. An alternative to microscopy is to use a rapid diagnostic test (RDT). Malaria RDT technology is attractive to health care settings lacking in human resources especially facing a high caseload of patients with suspected malaria, often including a high proportion of children under five years of age. These tests can be used by personnel including laypersons, and not necessarily laboratory technicians, after limited training. Malaria RDT results are on average available in less than 30 minutes.

The two types of RDT most often used belong to two groups according to the detected antigens: those detecting histidine-rich protein II (HRP2) and those detecting parasite specific lactate dehydrogenase (pLDH).

HRP2 antigen is one of the three histidine-rich proteins produced solely by trophozoites and young gametocytes of *Plasmodium falciparum*. RDTs based on the detection of HRP2 can only diagnose *Plasmodium falciparum *infections and thus cannot be used for the detection of *Plasmodium vivax *or other human malarias (*Plasmodium ovale*, *Plasmodium malariae*, and *Plasmodium knowlesi*, the latter only recently be detected in Asia [5]).

The malaria RDT most commonly used in the field by Médecins Sans Frontières (MSF) at present is Paracheck-Pf^®^, which belongs to the HRP2 group. Paracheck-Pf^® ^has widely proven its field reliability to detect *Plasmodium falciparum *in various continents, countries and settings [i.e. [6-8]]. However, a limitation of HRP2-based tests is their persistent false positivity after effective treatment of the infection. HRP2 is only slowly eliminated from the blood stream as it is expressed in the erythrocyte membrane [9]. This fact renders inconclusive a positive RDT result with a history of a recently treated infection, especially in areas of high transmission [i.e. [10-13]].

This is less of a problem with the pLDH- based tests. PLDH is an intracellular metabolic enzyme produced by all *Plasmodium *species that infect humans and is produced by sexual and asexual stages of the parasites. PLDH is cleared more quickly from the bloodstream than HRP2 after starting an effective treatment, as it is an enzyme only produced by viable parasites [i. e. [12,14-16]]. However, production of pLDH from gametocytes after elimination of asexual stages means some tests will still stay falsely positive for several days [17]. As pLDH-based tests are able to detect all human related *Plasmodium*, these RDTs could reduce the chance of patients being treated unnecessarily for diseases other than malaria or leave non-falciparum malaria untreated as it would be the case with a HRP2-based test.

Unfortunately, to date, the sensitivity of these tests, under field conditions (mainly using the OptiMal-IT test), has often been reported as lower than their HRP2 counterparts and falling below the internationally recommended standard of 90% [8,18,19]. However, two recent studies have shown more promising results with sensitivities over 90% demonstrated with CareStart™ two-line PAN-pLDH and CareStart™ three-line P.f/PAN-pLDH tests [12,20].

This study evaluated CareStart™ three-line P.f/PAN-pLDH test in comparison to Paracheck-Pf^®^. CareStart™ three-line P.f/PAN-pLDH was chosen for three reasons.

First, CareStart™ three-line P.f/PAN-pLDH is able to detect falciparum malaria as well as non-falciparum malaria. In a region where falciparum malaria is predominant but other species are also present, this test may contribute to a more differentiated diagnosis of malaria.

Second, pLDH-based tests are cleared more rapidly from the blood [12,14-16]. In regions with high malaria transmission, it is especially important that RDTs can be relied upon to test for new malaria infections shortly after effective treatment of the initial diagnosis.

Third, among pLDH-based tests CareStart™ recently showed its capacity to reach a sensitivity of over 90% [12,20].

Paracheck-Pf^® ^was chosen for comparison since it is currently in use in the MSF project in Sierra Leone and is considered to have a high sensitivity for *Plasmodium falciparum *[6-8,21]. Nevertheless, it has a relatively long false positivity of the test after effective treatment of the patient and only detects falciparum malaria [i.e. [11-21]].

There is an ongoing debate whether or not anti-malarial treatment should be given to children under five years of age only on the basis of clinical suspicion for malaria [4,22-24]. There is a trend towards laboratory-confirmed, usually RDT based, diagnosis [3,4]. However, due to the lack of evidence, opinions vary broadly. So far sensitivity and specificity studies for malaria RDTs, with one exception in the Democratic Republic of Congo [11], did not focus on children under five years of age. Operational research is urgently needed to provide evidence for the use of malaria RDTs in this age class.

The two main aims of the study were 1) to compare the sensitivity and specificity of CareStart™ three-line P.f/PAN-pLDH test and Paracheck-Pf^® ^to diagnose *Plasmodium falciparum *malaria in children under five years of age using Giemsa-stained blood smears as gold standard reference and 2) to assess the time required for false-positive CareStart™ three-line P.f/PAN-pLDH tests to become negative after successful treatment with ACT.

Secondary aims included assessing ease of use and inter-reader agreement of both malaria RDTs.

## Methods

### Study design

A prospective, single-blind evaluation of two RDTs compared to slide microscopy.

### Study site

The study was carried out in the Gerihun community health centre (CHC) in Bo district, one of five CHCs in which MSF is working. Bo district, situated in south-east Sierra Leone, is a hyperendemic malaria region where transmission is perennial. In 2008, of the 417,576 consultations in all MSF health facilities, 181,711 (43.5%) had uncomplicated malaria confirmed by RDT. Severe malaria is the principal cause of morbidity in the area, accounting in 2008 for 54.3% (3733/6875) of all admissions of children under five years of age in the paediatric department of the MSF-run referral hospital (MSF, internal source, 2008/2009). For its catchment population of 152,000 people MSF offers free malaria diagnosis, treatment and prevention in a setting of free primary heath care.

### Enrolment of study participants

All children under five years of age consulting Gerihun CHC for fever and/or history of fever in the previous 72 hours were screened for clinical suspicion of malaria according to routine CHC protocols. Children were included in the study if they satisfied all of the following screening criteria: age between two and 59 months, suspected malaria defined as fever (axillary temperature > 37.5°C), and/or history of fever in the 72 hours prior to attending Gerihun CHC, no signs of severe disease and/or clinical danger, no treatment for malaria administered within the previous four weeks, and signed, informed consent by responsible caregivers.

Patients were included for follow-up of the CareStart™ three-line P.f/PAN-pLDH test if they satisfied all of the following criteria in addition to the initial inclusion criteria: positive CareStart™ three-line P.f/PAN-pLDH test for at least one malaria species and positive malaria blood smear at Day 0 (day of inclusion), place of residence less than one hour walking distance from Gerihun CHC, and able to attend follow-up for up to 28 days.

### Sample size

To detect *Plasmodium falciparum *with a sensitivity of 90%, 138 blood smear positive patients were required (alpha error 0.05, precision 5%). Similar calculations applied to the specificity, so an additional 138 blood smear negative patients needed to be tested. To allow for an estimated 5% of patients with incomplete information, patients were continuously recruited until a minimum of 145 positive and 145 negative blood smears had been included. The study was not powered to evaluate detection of malaria species other than *Plasmodium falciparum*, or mixed infections that included *Plasmodium falciparum*.

A convenient sample size of 145 patients with a positive CareStart™ three-line P.f/PAN-pLDH test result and positive malaria blood smear at Day 0 (day of inclusion) was chosen to describe the time taken for the false-positive CareStart™ three-line P.f/PAN-pLDH tests to become negative.

### Study procedures

On the day of inclusion, demographic and clinical information were recorded, thick and thin blood smears prepared, and the two malaria RDTs (CareStart™ three-line P.f/PAN-pLDH test and Paracheck-Pf^®^) performed. Treatment was based on the results of the blood smear, taken as the gold standard reference diagnosis. All positive patients were treated for free according to the following treatment scheme recommended by World Health Organization (WHO), the national and MSF protocol: artesunate 4 mg/kg body weight and amodiaquine 10 mg/kg body weight once a day on Days 0-2. Patients eligible for the follow-up part of the study had all been treated with three doses of artesunate and amodiaquine, administered at the study site, and were observed over the first 30 minutes for immediate vomiting. Failure to complete the full course of treatment at the study site resulted in exclusion from the follow-up study part.

### Follow-up of CareStart™ three-line P.f/PAN-pLDH false-positive tests

Patients with a positive CareStart™ three-line P.f/PAN-pLDH test and a negative blood smear on the last day of treatment, Day 2, were followed from Day 2 with repeated CareStart™ three-line P.f/PAN-pLDH tests and blood smears at 7-day intervals until the CareStart™ three-line P.f/PAN-pLDH test results were negative or a maximum of 28 study inclusion days were reached.

### Evaluation of malaria rapid diagnostic tests (RDTs)

Both malaria RDTs were of traceable quality (standard supplier, used within the shelf life) and had a guaranteed history of proper storage and transport conditions. All RDTs were performed and interpreted according to the manufacturers' instructions.

1) CareStart™ three-line P.f/PAN-pLDH (batch number AI8IL, manufacturer's catalogue number G0121, AccessBio, New Jersey, USA) is an individually packaged test cassette, diagnosing *Plasmodium *infections by pLDH detection, distinguishing between *Plasmodium falciparum *and the other malaria species *Plasmodium vivax*, *Plasmodium malariae*, or *Plasmodium ovale*. It requires 5 μl of whole blood to be collected with a pipette provided by the test kit. Test results need to be read after 20 minutes.

2) Paracheck-Pf^® ^(batch numbers 31930 and 31932, manufacturer's catalogue number 30301025, Orchid Biomedical System, Goa, India) is also an individually packaged test cassette, diagnosing *Plasmodium falciparum *infections by HRP2 detection. It requires 5 μl of whole blood to be collected with a sample applicator provided by the test kit. Test results need to be read after 15 minutes.

Test results were read by two independent readers, who received training before the beginning of the study. The readers were taught the procedure and interpretation of the RDT results. The study did not start until the study supervisor was satisfied that the readers were familiar with handling and reading of both tests. Preparation and reading of the RDTs were continually reviewed under the observation of the study supervisor. The first reading was performed at the time specified by the manufacturer. The person doing the first reading was a trained laboratory technician and also prepared the two RDTs beforehand. As the two manufacturers provided no maximum time before reading the results, the second reading was performed less than 10 minutes after the first. The second reading was done by a trained layperson. The readers were blinded to each other's results and to that of the blood smear. For each RDT the result was classified as negative, positive (differentiating for the CareStart™ three-line P.f/PAN-pLDH test *Plasmodium falciparum *or other *Plasmodium *species) or invalid. All tests without a control line were considered invalid by the first reader and were repeated.

### Laboratory procedures

Thick and thin blood smears and RDTs were performed from the same finger-prick of blood. Thick and thin smears were prepared on the same slide and were air-dried. The thin blood smears were fixed with methanol and the thick smears left unfixed. Each slide was then stained with 10% Giemsa solution for ten minutes. All blood smears were examined microscopically under oil immersion (× 1,000 magnification). The thick smears were used for diagnosis of *Plasmodium *species and for parasite-density counting. Smears were considered negative if no parasites were seen in 100 oil-immersion fields. For positive smears, the number of parasites was counted against 200 white blood cells (WBC), or 500 WBC for low-density infections. Parasite density was calculated assuming 8,000 WBC per microlitre. The thin smears were examined to confirm the parasite species for positive samples. Gametocyte presence or absence on all slides was also recorded, and species identification and gametocyte-density counts (number of gametocytes/1,000 WBC) performed.

All inclusion slides were double-read, blinded, by experienced technicians (one in the MSF project in Sierra Leone and one in MSF Austria that has staff with a special focus on laboratory quality). A third reading was performed in case of discordance, such as positive/negative discordance for asexual stages, species discordance for asexual stages, asexual density discordance (difference in parasitaemia ≥50%), and/or positive/negative gametocyte discordance. Third readings were performed by the Department of Infectious and Tropical Diseases at the London School of Hygiene and Tropical Medicine and were taken as the definitive results for these slides being disconcordant between the first and the second reader.

### Assessment of ease of use of RDTs

At the end of the field part of the study, the ease of use of both RDTs was compared using quantitative (storing conditions) and qualitative criteria. The study laboratory technicians who performed the RDTs were asked to rank the tests independently in order of preference where "2" corresponded to their most preferred test and "0" to their least preferred in each of the following categories: Ease and safety of taking blood, ease of adding reagents, ease of interpretation and quality of instructions. The trained laypersons who were performing second readings of tests were asked to rank the tests independently based on the ease of interpretation, and for any additional comments where "2" corresponded to their most preferred test and "0" to their least preferred.

### Data management and analysis

All data were recorded directly on an individually numbered case report form. Data were entered into EpiData 3.0 software (The EpiData Association, Odense, Denmark). Data cleaning was done to check for inconsistencies in data entry and responses. Data analysis was conducted using Stata 8.0 (Stata Corporation, College Station, Texas, USA) and SPSS 11.0 (SPSS. Chicago, USA).

Baseline characteristics (demographic, clinical, and parasitological) were analysed using descriptive statistics (means, inter-quartile ranges and values). Sensitivity was defined as the proportion of true malaria cases (positive blood smears) that were correctly identified by positive RDTs. Specificity was the proportion of true negative malaria cases (negative blood smears) that were correctly identified by negative RDTs. Positive predictive value was the proportion of true malaria cases (positive blood smears) among the total number of positive RDTs. Negative predictive value was the proportion of true negative malaria cases (negative blood smears) among the total number of negative tests.

Sensitivity, specificity, positive and negative predictive values of each test were calculated for RDTs performed at the day of inclusion and estimated using microscopy as the reference standard. Sensitivity, specificity, positive and negative predictive values were also stratified by category of parasitaemia (<100, <1,000, ≥1,000 parasites/μl). The first reading of the RDTs was used for these calculations. A 95% confidence interval (95% CI) was given for each parameter. Proportions (paired data) were compared using the McNemar test. Agreement between readers of each RDT was assessed using the kappa coefficient. A Kappa coefficient greater than 0.8 was considered as a measure of good agreement.

### Ethical issues

Approval was received from the Ethics Review Board of MSF and the Research and Ethics Committee of the Ministry of Health and Sanitation of Sierra Leone. Informed, written consent was sought from responsible caregivers of all children participating in the study. The caregivers were provided with an information sheet and had the study purpose explained in their own language by the study personnel (Mende, Creole or English). Participation was entirely voluntary.

## Results

### Demographic and parasitological characteristics of study patients

Between 17 March and 15 July, 2008, 343 participants under five years of age were included in the study. Baseline characteristics of the study population are shown in Table 1. None of the children's caregivers refused to participate in the study. Among the 343 study participants, 167 were eligible for follow-up of CareStart™ three-line P.f/PAN-pLDH false-positive tests at Day 0 (day of inclusion). Of the 176 study participants not eligible for follow-up at Day 0 (day of inclusion), 167 had a CareStart™ three-line P.f/PAN-pLDH negative test in combination with a negative blood smear, seven had a CareStart™ three-line P.f/PAN-pLDH false-positive test in combination with a negative blood smear, and two failed to complete the full course of anti-malarial treatment under supervision. Of the 343 patients tested at Day 0 of the study, 46.7% (169/343) had malaria positive blood smears. The only species detected in the positive blood smears was *Plasmodium falciparum*. Parasitological characteristics of smear-positive study participants are given in Table 1. A high parasitaemia, defined as ≥ 1,000 parasites/μl, was seen in 82.8% (140/169) of the study patients. Of these, 49.3% (69/140) had ≥ 50,000 parasites/μl. Seventeen study patients had a positive blood smear with both asexual stages and gametocytes (10.1%).

**Table 1:** Baseline characteristics of all study patients and parasitological characteristics of slide-positive participants attending Gerihun community health centre in Bo district, Sierra Leone, Africa, 2008

Baseline characteristics (N = 343)	
	
Age:	
Mean, median in months (IQR^#^)	23,20 (9-36)
Range in months (Minimum, Maximum)	2,58
2 - 23 months (%)	252 (73.4%)
24 - 59 months (%)	91 (26.6%)
Gender ratio (male/female)	1.1 (177/166)
Median weight in kg (IQR^#^)	9.5 (7.8-11.8)
Median temperature on day 0 (IQR^#^)	37.8 (37.6-38.4)
Temperature > 37.5° C on day 0 (%, n/N)	75.2 (258/343)
Parasitological characteristics (N = 169)	
	
Asexual parasitaemia density range (parasites/μl)	1 - 2136000
Median of asexual parasitaemia density (parasites/μl)	264000
Inter-quartile parasitaemia density range (parasites/μl)	2620-79921
Gametocyte carriage for *Plasmodium falciparum *(n, %)	17, 10.1%
Median number of gametocyte density^† ^(IQR^#^)	3 (2-13)

^# ^IQR: inter-quartile range

^† ^Number of gametocytes per 1000 white blood cells

### Accuracy of RDTs

There was no significant difference between the sensitivity of CareStart™ three-line P.f/PAN-pLDH (168/169, 99.4%) and Paracheck-Pf^® ^(167/169, 98.8%) (Table 2). CareStart™ three-line P.f/PAN-pLDH was significantly more specific than Paracheck-Pf^® ^with 96.0% (167/174) and 74.7% (130/174), respectively (p < 0.001). Stratification by level of parasitaemia (<100 versus ≥100 parasites/μl and <1,000 versus ≥1,000 parasites/μl) did not show any differences in terms of sensitivity and specificity results of the two RDTs. The only false negative result for CareStart™ three-line P.f/PAN-pLDH test had a parasitaemia of 1-2 parasites/μl. The two study persons with false negative results for Paracheck-Pf^® ^test had a parasitaemia of 288,000 and 580,000 parasites/μl.

**Table 2:** Accuracy of CareStart™ three-line P.f/PAN-pLDH and Paracheck-Pf^® ^malaria rapid diagnostic tests for the detection of *Plasmodium falciparum* in patients attending Gerihun community health centre in Bo district, Sierra Leone, Africa, 2008

		CareStart™ 3 line P.f/PAN-pLDH	Paracheck-Pf^®^
Sensitivity	% (95% CI^#^)	99.4 (96.8 - 100.0)	98.8 (95.8 - 99.8)
	n/N	168/169	167/169
Specificity^Ψ^	% (95% CI^#^)	96.0 (91.9 - 98.4)	74.7 (67.6 - 81.0)
	n/N	167/174	130/174
PPV^†^	% (95% CI^#^)	96.0 (91.9 - 98.4)	79.2 (73.0 - 84.4)
	n/N	168/175	167/211
NPV^‡^	% (95% CI^#^)	99.4 (96.7 - 100.0)	98.5 (94.6 - 99.8)
	n/N	167/168	130/132
Kappa coefficient	% (95% CI^#^)	0.95 (0.92 - 0.98)	0.99 (0.98 - 1.0)

^# ^Confidence interval

^Ψ ^p < 0.001

^† ^Positive predictive value

^‡ ^Negative predictive value

### Follow-up of false-positive CareStart™ three-line P.f/PAN-pLDH test after successful anti-malarial treatment

Out of the 167 study patients eligible for follow-up, 12 were lost to analysis. Five defaulted at Day 1, one had an incorrect artesunate and amodiaquine intake on Day 2, one needed referral to the hospital due to deteriorating health status, and five blood slides from Day 2 were broken during transport and consequently were unreadable. Altogether, 155 study patients were included in the follow-up analysis. There were no positive blood smears for any of the 155 study patients on any of the follow-up days. A positive CareStart™ 3 line P.f/PAN-pLDH test during that period was considered as a false-positive result.

**Figure 1:**
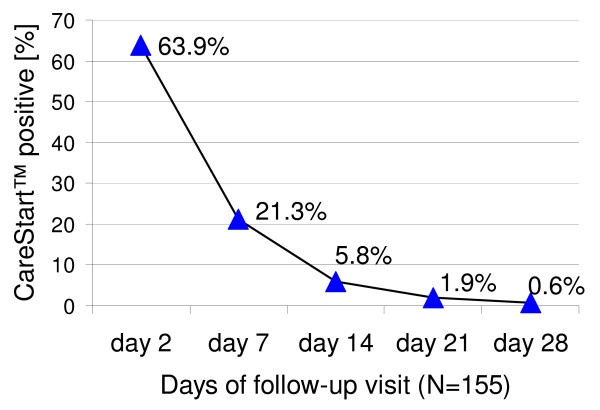
**Proportion of false-positive CareStart™ three-line P.f/PAN-pLDH tests on each follow-up visit of study patients attending Gerihun community health centre in Bo district, Sierra Leone, Africa, 2008**.

On Day 2 of follow-up, 63.9% (95% CI 56.0-71.0, 99/155) of the study participants had a CareStart™ three-line P.f/PAN-pLDH false-positive test. Similarly, on day seven, 21.3% (95% CI 15.6-28.4, 33/155), on day 14, 5.8% (95% CI 3.0-10.7, 9/155), on day 21, 1.9% (95% CI 0.6-5.5, 3/155) and on day 28, 0.6% (95% CI 0.1-3.6, 1/155) had false-positive tests (Figure 1). The median time for CareStart™ three-line P.f/PAN-pLDH test to become negative during the follow-up was seven days (mean 7 days, inter-quartile range 2-7 days).

### Ease of use and reliability of RDTs

Remarks made were that the two RDTs were equally safe for personnel to handle and easy to use in terms of adding blood and reagents. Both the control and the positive test lines were equally readable in both RDTs. The instruction sheet provided with Paracheck-Pf^® ^included more detailed and comprehensive information than that provided with the CareStart™ three-line P.f/PAN-pLDH test. This was true for both general background information and specific practical information about the test and its component parts. For example, the Paracheck-Pf^® ^instruction sheet informed the user of the presence of the packet of silica gel and that a colour change in this gel from blue to pink signified that the test had been exposed to compromising levels of humidity which invalidate the test. The CareStart™ three-line P.f/PAN-pLDH tests were packed with a small tablet, which had no identification markings. There was no information about this tablet in the instruction sheet provided. Both tests were simple to store with no cold chain requirement for either test. In terms of storage capacity, one box of 75 CareStart™ three-line P.f/PAN-pLDH tests was approximately the same size as one box of 25 Paracheck-Pf^® ^tests. However, the CareStart™ three-line P.f/PAN-pLDH box did not include lancets or alcohol swabs. Both tests had equal scores in ranking. The number of invalid tests was 0.6% (4/605) for CareStart™ three-line P.f/PAN-pLDH and 0.3% (1/343) for Paracheck-Pf^®^.

The control and the positive test lines were equally readable for both RDTs. Kappa coefficient for the inter-reader reliability for both RDTs tests was above 0.9 (Table 2).

## Discussion

Both malaria RDTs, CareStart™ three-line P.f/PAN-pLDH and Paracheck-Pf^®^, were highly sensitive for the detection of falciparum malaria monoinfections in children under five years of age. When a parasitaemia of more than 100 parasites per microlitre was considered, both tests were >95% sensitive for the detection of *Plasmodium falciparum*, meeting the criteria set out by WHO [19,25]. A literature search produced only two other published studies assessing the sensitivity of CareStart™ RDTs to detect *Plasmodium falciparum *malaria in Africa. Both studies used the two-line test and did not exclusively focus on children under five years of age. They included a field study in Uganda, which found sensitivity of the CareStart™ two-line PAN-pLDH test to be 95.6% [12], and a study in Madagascar, where sensitivity of CareStart™ two-line PAN-pLDH was 97% [20]. In a survey carried out recently in Myanmar, CareStart™ three-line P.f/PAN-pLDH tests had a sensitivity of 94.7% [26]. The results of these studies confirm the high sensitivity of CareStart™ tests and make this pLDH-based test a strong candidate for consideration when selecting the most suitable RDT in projects in hyperendemic falciparum malaria areas.

Both malaria RDTs were very reliable, having less than one percent invalid tests. Inter-reader reliability was equally good, indicating that wherever the tests are used, readers will be able to give trustworthy results. Both tests were perceived by the study team as similar in terms of ease of use. Therefore, neither RDT was preferable based on these characteristics.

Heat-stability has been a major concern for pLDH tests, especially under field conditions [27]. Due to very basic field conditions no stability testing was carried out in this study. However in a study carried out in Myanmar CareStart™ three-line P.f/PAN-pLDH tests remained stable for up to 90 days at 35°C [26]. In the 2009 WHO's evaluation of RDTs performance, the heat stability of CareStart™ two-line PAN-pLDH remained stable for up to 60 days at 4°C, 35°C and 45°C [28]. Thus, stability of the CareStart™ three-line P.f/PAN-pLDH seems to be satisfactory.

The major advantage of pLDH-based tests over HRP2-based tests is their ability to rapidly become negative after effective treatment with an anti-malarial. This is in contrast to Paracheck-Pf^® ^that remains false-positive for up to four weeks after successful treatment. In the Democratic Republic of Congo, 73.5% of effectively treated children were still Paracheck-Pf^® ^false-positive at Day 35 [11]. In Uganda, 69.7% of successfully treated patients remained Paracheck-Pf^® ^false-positive at Day 14 [12]. In southern Vietnam, the Paracheck-Pf^® ^false positivity rate was around 30% at Day 28 of follow-up [21]. The first results seen with CareStart™ were very promising - in Uganda 14 days after treatment only 10% of the CareStart™ 2 line PAN-pLDH tests remained false-positive. In that study, the follow-up stopped at Day 14 [12]. This rapid return to negativity was further confirmed in the present study. CareStart™ 3 line P.f/PAN-pLDH became negative after a median of seven days. On the 14^th ^day of follow-up less than 6% of the study patients still tested false-positive, approximately half the percentage found false-positive during the Uganda study at the same follow-up day. At Day 28, less than one percent of the study patients were still false-positive. Especially in a hyperendemic setting, it is extremely important to have a short parasite antigen clearance time after parasites are removed. This characteristic allows health personnel to confidently interpret positive RDT results without concerns over possible false-positive results from previously treated infections.

At the time this study was planned (early 2008), WHO did not endorse any particular RDT, but listed products submitted to the WHO malaria RDT product testing programme [29]. By April 2009, the outcome of the first round of product testing malaria antigen-detecting RDTs was made public [28]. Both of the RDTs test manufacturers in this study were invited to submit up to three tests for evaluation. AccessBio submitted the CareStart™ two-line PAN-pLDH test while Orchid Biomedical System participated with Paracheck-Pf^®^. All tests were evaluated using prepared blood panels of cultured *Plasmodium falciparum *parasites and patient-derived *Plasmodium falciparum *and *Plasmodium vivax *parasites, and a parasite-negative panel.

In the WHO evaluation, CareStart™ two-line PAN-pLDH performed better than Paracheck-Pf^® ^in terms of detection rate of parasites, inter-reader agreement, and heat stability. However, they both showed an equivalent high level of detection at a high parasite density (2,000 or 5,000 parasites/μL). It has to be noted that the WHO testing was an evaluation of the performance under laboratory conditions. In contrast, this study was carried out in the field and was the first to do so focusing on children under five years of age under these conditions. Future procurement decisions should be based on WHO test results, but need to take into consideration local conditions of malaria transmission and illness where the test will be used. Nevertheless, to centralise RDT product testing *in vitro *is a very welcome initiative from WHO to facilitate the evaluation of reliable RDTs in the field.

There are some potential limitations in generalizing this promising CareStart™ three-line P.f/PAN-pLDH test as a suitable candidate for malaria diagnosis. The study team was well-trained and constantly supervised in test preparation and interpretation. This might not be the case in all health facilities using this technology. Also, the study cohort only had *Plasmodium falciparum *malaria species to detect. CareStart™ three-line P.f/PAN-pLDH tests might perform less satisfactorily in settings where other malaria species predominate, which was the case in a study in Myanmar [26]. As the study participants all had a mono-infection with *Plasmodium falciparum *no conclusions can be drawn in terms of sensitivity of CareStart™ three-line P.f/PAN-pLDH test for the other *Plasmodium *infections. This confirms the need to carry out RDT assessments in different geographical areas and with different types and levels of transmission in order to find the most suitable test for each region.

Several studies demonstrate inconsistencies in blood smear reading and, therefore, possibly doubtful results for sensitivity and specificity of malaria RDTs [30,31]. Some studies even suggest that RDTs perform better than microscopy under routine conditions [32] or at parasite densities below the threshold for detection by microscopy [33]. Because blood smears were used as the gold standard reference to compare both RDTs under evaluation, extra emphasis was placed on quality control of the blood smears. All slides were read twice, by two independent well-trained microscopists, who were blinded to both the RDTs results and the findings of their counterpart. In addition, all slides with disconcordant results were read a third time by the Department of Infectious and Tropical Diseases at the London School of Hygiene and Tropical Medicine in London.

## Conclusions

Before choosing a malaria RDT for use in the field it must fulfil a number of requirements. In this study CareStart™ three-line P.f/PAN-pLDH fulfilled all the requested criteria: (1) Sensitivity for the diagnosis of falciparum malaria was more than 95% with a parasitaemia greater than 100/μl. (2) All further accuracy parameters (specificity, positive and negative predictive values) were above 90%. (3) The test had an excellent inter-reader agreement and was reliable, easy to use and interpret. (4) False positivity decreased rapidly after successful anti-malarial treatment and became, on average, negative after seven days.

This study confirms the value of RDTs for use in children under five years of age by refuting the idea that RDTs may miss a significant number of diagnoses in this age group. Widespread use of RDTs should improve diagnostic accuracy of malaria in the field and should be adopted by malaria control programmes. These results confirm that, in terms of accuracy, CareStart™ three-line P.f/PAN-pLDH test can be used as an alternative to Paracheck-Pf^® ^in hyperendemic settings where *Plasmodium falciparum *malaria is the predominant species.

On the basis of this study, policy makers and governmental and non-governmental health providers can consider CareStart™ three-line P.f/PAN-pLDH test as a valuable tool for diagnosing falciparum malaria in children under five years of age, especially in more remote areas with hyperendemic conditions. This supports the wider use of RDTs to accurately diagnose malaria and avoid over or under treatment.

## Competing interests

The authors declare that they have no competing interests.

## Authors' contributions

SG was the principal investigator of the study. She designed the study and collected data in the field. She did the study analysis, interpretation of data and wrote the paper. SD, AM and SB supported the principal investigator considerably in carrying out the field work. MDS and JM did a critical revision of the paper. All authors read and approved the final manuscript.
